# Implementing within‐cross genomic prediction to reduce oat breeding costs

**DOI:** 10.1002/tpg2.20004

**Published:** 2020-03-17

**Authors:** Greg Mellers, Ian Mackay, Sandy Cowan, Irene Griffiths, Pilar Martinez‐Martin, Jesse A. Poland, Wubishet Bekele, Nicholas A. Tinker, Alison R. Bentley, Catherine J. Howarth

**Affiliations:** ^1^ The John Bingham Laboratory NIAB Cambridge United Kingdom; ^2^ IMplant Consultancy Ltd. Chelmsford United Kingdom; ^3^ Institute of Biological, Environmental and Rural Sciences, Plas Gogerddan Aberystwyth University Aberystwyth United Kingdom; ^4^ Wheat Genetics Resource Center, Department of Plant Pathology Kansas State University Manhattan KS USA; ^5^ Ottawa Research and Development Centre, Agriculture and Agri‐Food Canada Ottawa Canada

## Abstract

A barrier to the adoption of genomic prediction in small breeding programs is the initial cost of genotyping material. Although decreasing, marker costs are usually higher than field trial costs. In this study we demonstrate the utility of stratifying a narrow‐base biparental oat population genotyped with a modest number of markers to employ genomic prediction at early and later generations. We also show that early generation genotyping data can reduce the number of lines for later phenotyping based on selections of siblings to progress. Using sets of small families selected at an early generation could enable the use of genomic prediction for adaptation to multiple target environments at an early stage in the breeding program. In addition, we demonstrate that mixed marker data can be effectively integrated to combine cheap dominant marker data (including legacy data) with more expensive but higher density codominant marker data in order to make within generation and between lineage predictions based on genotypic information. Taken together, our results indicate that small programs can test and initiate genomic predictions using sets of stratified, narrow‐base populations and incorporating low density legacy genotyping data. This can then be scaled to include higher density markers and a broadened population base.

**Abbreviations**
BLUP: Best linear unbiased prediction
CV: Cross‐validation
DArT: Diversity Array Technology
DiPR: Differentially penalized ridge regression
GBS: Genotyping‐by‐sequencing
GEBV: Genomic estimated breeding value
GS: Genomic selection
LD: Linkage disequilibrium
MCCV: Monte Carlo cross‐validation
RIL: Recombinant inbred line
RR‐BLUP: Ridge regression‐BLUP
SSD: Single‐seed descent
SNP: single nucleotide polymorphism

## 1 INTRODUCTION

The adoption of affordable genetic markers in breeding programs has expanded the use of accelerated, genomic‐based breeding approaches from genome‐wide information (Lorenzana & Bernardo, 2009; Morrell, Buckler, & Ross‐Ibarra, 2011). Genomic selection (GS) based on the selection of individuals using a genomic estimated breeding value (GEBV) can enable faster, more intense and more accurate selection (Heffner, Sorrells, & Jannink, 2009; Meuwissen, Hayes, & Goddard, 2001).

Ongoing research in crops has progressed beyond improving prediction accuracy and now centers on how best to employ GS within breeding programs (Arruda et al., 2015; Bassi, Bentley, Charmet, Ortiz, & Crossa, 2015; Jarquín et al., 2016; Norman, Taylor, Edwards, & Kuchel, 2018; Vivek et al., 2017), although the transition to practical implementation in small programs remains a challenge (Voss‐Fels, Cooper, & Hayes, 2019). This is predominantly due to the initial expense of genotyping existing germplasm. Recent work has shown that a modest number of markers can be sufficient to achieve accurate predictions in small populations with high linkage disequilibrium (LD; Gonen et al., 2018; Norman et al., 2018). It is necessary to consider how to gain value from the upfront cost of genotyping material that may not be progressed within an active breeding program. Additionally, training sets should logically be developed from breeding lines or populations (Akdemir & Isidro, 2019; Akdemir, Sanchez, & Jannink, 2015; Asoro, Newell, Beavis, Scott, & Jannink, 2011; Isidro et al., 2015; Ou & Liao, 2019; Rincent et al., 2012). Therefore, in small programs, the gradual generation and use of genotypic data in narrow‐based populations can support the longer‐term adoption of GS.

In a biparental crossing scheme, high levels of LD can be exploited to minimize genotyping cost. In cultivated oat (*Avena sativa* L.), high levels of long‐range LD (Esvelt Klos et al., 2016) and large haplotype blocks have been reported (Bekele, Wight, Chai, Howarth, & Tinker, 2018), along with clustering of Diversity Array Technology (DArT) markers (Tinker et al., 2009). Selfing limits the amount of recombination per generation, reducing LD dissipation, and increasing genetic variance between the resulting recombinant inbred lines (RILs), thus promoting the emergence of superior transgressive segregants (McClosky, LaCombe, & Tanksley, 2013). These factors make within‐cross GS feasible to assess performance relative to the other lines in the same (rather than different) subpopulations (Asoro et al., 2013; Gonen et al., 2018; Gorjanc et al., 2017a, 2017b). Previous work has tested biparental prediction approaches via simulation in a maize (*Zea mays*) genome (McClosky et al., 2013), concluding that gains attributable to selfing are achievable across different population sizes, trait heritabilities, and selection intensities. Lorenzana and Bernardo (2009) evaluated GS in two double haploid biparental barley (*Hordeum vulgare*) populations using historical trial data on production and quality traits and 223 polymorphic markers. They reported that the simple and computationally efficient best linear unbiased prediction (BLUP) approach was ideally suited to biparental GS. Additionally, extensive LD and large linkage blocks meant that fewer markers were needed for accurate predictions (Lorenzana & Bernardo, 2009).

Cultivated hexaploid oat is a cereal crop used to produce grain in temperate regions and forage in the subtropics (Hoffman, 1995). The allopolyploid oat genome is large (12.5 gigabases) and highly repetitive, making the large‐scale adoption of genomics‐based breeding methods difficult (Yan et al., 2016). Recent advances in genomic resources (e.g., Chaffin et al., 2016; Huang, Poland, Wight, & Jackson, 2014) mean GS is now more tractable for uptake within oat breeding programs. Previous work to evaluate the application of GS in elite‐cultivated North American oat lines for both production and quality traits demonstrated that GS could be effective even at modest marker density (∼every 2cM; Asoro et al., 2011), although no plateau was reached with low density DArT marker numbers. Comparison of GS to traditional phenotypic and marker‐assisted selection for the complex quality trait β‐glucan showed that the benefits of GS could be realized based on a per cycle basis via the scaling of selection to two cycles per year (Asoro et al., 2013). More recently, Bekele et al. (2018) described the prediction of heading date in a large cultivated oat panel, reporting a minimal increase in accuracy from increasing marker density. However, their results showed that prediction from genotyping‐by‐sequencing (GBS) derived single nucleotide polymorphisms (SNPs) gave higher prediction accuracy than using tag‐level haplotype markers (Bekele et al., 2018).

Here we report the implementation of genomic prediction within a biparental cross between two cultivated winter oat varieties, ‘Buffalo’ and ‘Tardis’. The population has been previously used to update the oat consensus map based on GBS‐derived, tag‐level haplotypes (Bekele et al., 2018). In this study the population was stratified for both genotyping (at the F_2_ and F_7_ generation) and phenotyping (segregated at the F_3_ generation with one stream of material progressed to field assessment and the other undergoing rapid single seed descent [SSD] to the F_7_ generation). Using low‐coverage genotypic information in the early generation, we investigate the recovery of missing phenotypes via genomic prediction, which is required for accurate representation of true phenotypic value and variance. We also extend this to the F_7_ generation to test prediction of missing yield data. We demonstrate that using mixed marker data is feasible—with both low cost dominant markers and more expensive co‐dominant markers integrated to improve accuracy.

**Core Ideas**

- Predictions based on low coverage genotyping can recover missing phenotypes in early generations.
- Mixed data types can be effectively integrated to improve prediction accuracy in oat.
- Differentially penalized regression can optimally weight mixed data.

## 2 MATERIALS AND METHODS

### 2.1 Plant material, genotyping and phenotyping

An F_2_ mapping population of 194 individuals was produced from a cross between the two winter oat varieties ‘Buffalo’ and ‘Tardis’ at Aberystwyth University, United Kingdom. The population was created to capture key differences between the parents; ‘Buffalo’ is a dwarf variety with low kernel content and small grains and ‘Tardis’ is a conventional‐height variety with high kernel content and large grains. The DNA was extracted from the seedling leaves of F_2_ plants and the parents using a QIAGEN DNeasy 96 Plant Kit (QIAGEN, Crawley, United Kingdom) and genotyped using 121 polymorphic microsatellites (Dumlupinar et al., 2016; Jannink & Gardner, 2005; Li, Rossnagel, & Scoles, 2000; Pal, Sandhu, Domier, & Kolb, 2002; Wight, Yan, Fetch, Deyl, & Tinker, 2010; Wu, Zhang, Chen, & He, 2012) and with the oat DArT array (Tinker et al., 2009; Diversity Arrays Technology Pty, Canberra, Australia) which identified a further 424 polymorphic (dominant) loci.

**FIGURE 1:**
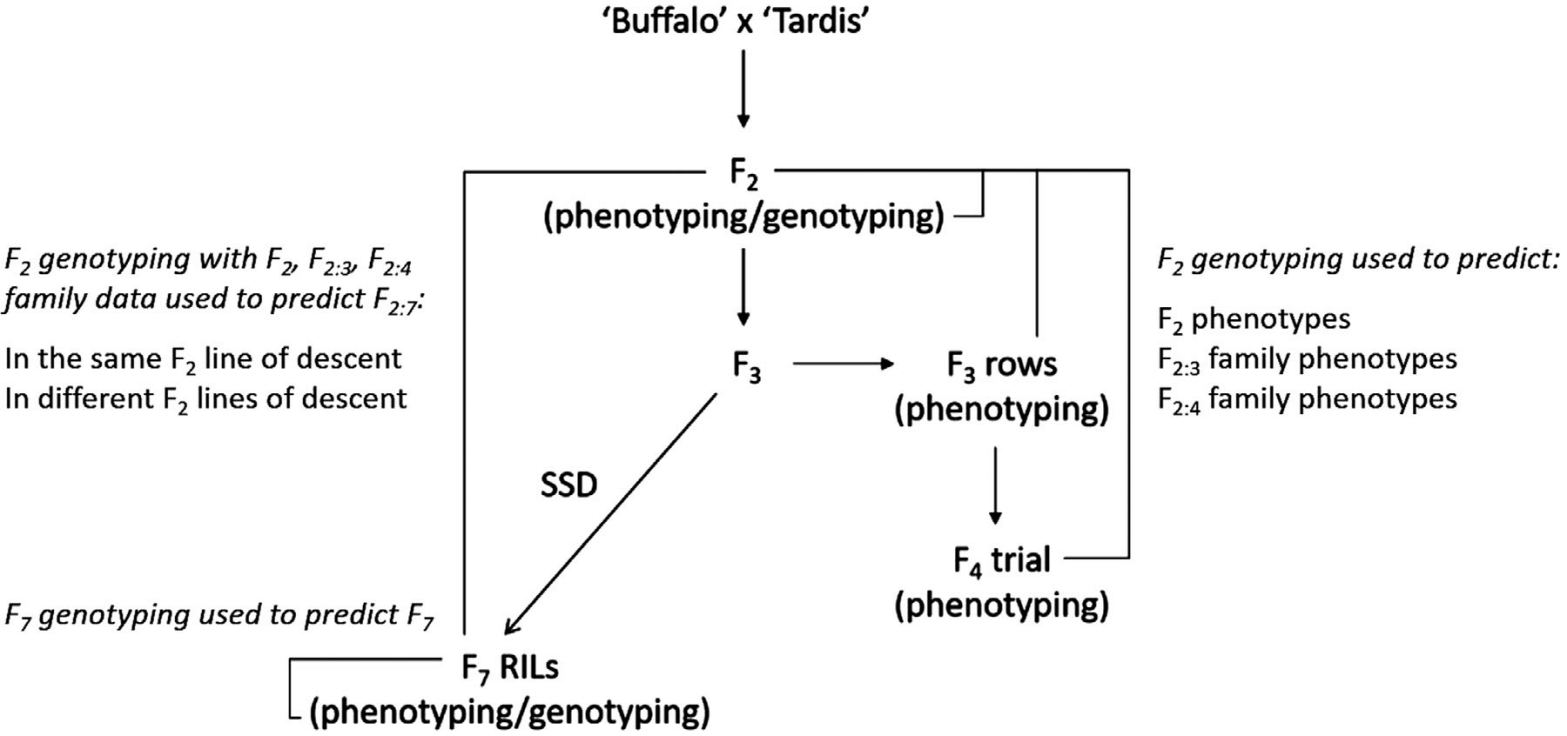
The stratification of within‐population advance of material in the ‘Buffalo’ × ‘Tardis’ population, including derivation of phenotyping and genotyping data used in this study

From each F_2_ plant, families of F_3_ seed were harvested and multiplied in the field to produce F_4_ bulks to enable sufficient seed for replicated field trials. Each F_2_‐origin plant therefore defines a lineage with the resulting progeny forming a family and F_3_ and F_4_ genotypes are inferred from the F_2_. A RIL mapping population of 227 individuals was derived by SSD from individual seeds of the F_3_ plants, giving a slightly larger number of individuals than the initial 194‐line F_2_ population. The population size was increased by selecting single seeds from individual F_3_ dwarf and tall plants. Progeny were advanced through SSD to the F_7_ generation where leaf material was sampled for DNA extraction as previously described (Figure 1). In addition to microsatellites and DArT markers, GBS libraries were constructed following the oat protocol developed and described by Huang et al. (2014) and processed as reported in Bekele et al. (2018). In this analysis, 1,046 markers were used for the RILs, and between the F_2_ and RIL datasets there were 401 common markers, of which 100 were codominant and 301 were dominant. Genotype calls and map locations are integrated into The Triticeae Toolbox oat platform (http://triticeaetoolbox.org/oat/genotyping) as reported in Bekele et al., 2018. Stratification of the population for both phenotyping and genotyping is summarized in Figure 1. Phenotypic assessment for production‐related traits (maturity, ear emergence, Internode 1 length, kernel content, panicle length, panicle extrusion, winter hardiness, height, grain yield, mildew, hullability, grain length, grain width, and grain area) was conducted in either the field or polytunnel at the F_2_ (2005), F_3_ (2006), and F_4_ (2007–2010) generation. In addition, the F_7_ RILs were phenotyped (2010–2014) for both the production characteristics (as previously) and the quality trait grain β‐glucan content at the RIL (F_7_; 2010–2014) generation (Table 1). All field trials were conducted in Aberystwyth, United Kingdom (52.43 lat, 4.02 long) and used standard pre‐emergence and early spring weed control with no fungicides or growth regulators applied. Nitrogen fertilizer (70 kg ha^−1^) was applied in a split dose at GS31 and GS35 (Zadoks, Chang, & Konzak, 1974). The traits were assessed using a range of standard phenotyping methods, summarized in Supplemental Table S1. The number of individuals phenotyped for each trait varied, and data was averaged across trial entries to derive phenotypic means (Table 1).

**TABLE 1:** Within generation predictions of traits assessed on the ‘Buffalo’ × ‘Tardis’ population with different generations of phenotyping (Gen^P^) and genotyping (Gen^G^) assessed in a range of years in field (F) or polytunnel (PT) trials. The ridge regression best linear unbiased predictor (RR‐BLUP) predictions are made across the full set of available lines (All), and within *Dw6* classes (Tall, Dwarf). SD, standard deviation

						Trait value		RR‐BLUP prediction		
Trait	Gen^P^	Gen^G^	Year assessed	Trial	*n*	Mean	SD	All	Tall	Dwarf
Internode 1 length, cm	F_2_	F_2_	2005	F	180	25.97	10.92	0.79	0.21	0.10
	F_4_	F_2_	2007, 2008	F	92	34.81	10.06	0.34	0.25	0.00
	F_7_	F_7_	2011, 2014	PT	227	36.36	12.51	0.83	‐	‐
Kernel content, %	F_4_	F_2_	2007, 2008, 2010	F	180	63.53	3.99	0.60	0.40	0.26
	F_7_	F_7_	2012	F	156	62.60	4.60	0.66	‐	‐
Maturity, d after 1 April	F_4_	F_2_	2008	F	180	112.12	1.22	0.33	0.32	0.33
	F_7_	F_7_	2013	F	156	95.96	1.62	0.79	‐	‐
Mildew	F_3_	F_2_	2006	F	180	0.54	1.29	0.40	0.23	0.40
	F_7_	F_7_	2011	PT	227	0.42	0.50	0.72	‐	‐
	F_7_	F_7_	2014	PT	91	0.53	0.50	0.86	‐	‐
Panicle extrusion, cm	F_4_	F_2_	2007, 2008	F	92	8.47	9.98	0.35	0.24	0.00
	F_7_	F_7_	2011	PT	227	12.02	12.42	0.87	‐	‐
Winter hardiness	F_3_	F_2_	2006	F	148	8.10	0.58	0.09	0.10	0.10
	F_4_	F_2_	2007–2009	F	180	7.28	0.44	0.70	0.60	0.67
	F_7_	F_7_	2011	F	184	1.81	1.13	0.47	‐	‐
	F_7_	F_7_	2012	F	227	7.29	0.64	0.77	‐	‐
Grain yield, t ha^−2^ at 85% dry matter	F_4_	F_2_	2007, 2008, 2010	F	180	1.07	0.34	0.63	0.00	0.30
	F_7_	F_7_	2014	F	227	4.91	2.19	0.66	‐	‐
Ear emergence, d after 1 April	F_2_	F_2_	2005	F	180	61.96	3.62	0.64	0.00	0.44
	F_4_	F_2_	2007, 2008, 2010	F	87	66.42	1.94	0.79	0.34	0.00
	F_7_	F_7_	2010, 2011	PT	227	78.65	16.60	0.72	‐	‐
	F_7_	F_7_	2010–2013	F	227	70.44	5.23	0.71	‐	‐
Height, cm	F_2_	F_2_	2005	F	180	101.38	28.00	0.81	0.00	0.44
	F_4_	F_2_	2007, 2008, 2010	F	180	113.59	19.81	0.88	0.00	0.76
	F_7_	F_7_	2010–2014	F	222	110.60	27.13	0.88	‐	‐
	F_7_	F_7_	2011, 2014	PT	222	137.68	32.56	0.89	‐	‐
Grain length, mm	F_4_	F_2_	2008	F	177	11.10	0.50	0.45	0.54	0.42
	F_7_	F_7_	2013	F	150	13.22	0.70	0.61	‐	‐
Grain width,	F_4_	F_2_	2008	F	177	3.40	0.10	0.61	0.50	0.59
	F_7_	F_7_	2013	F	150	3.07	0.14	0.65	‐	‐
Hullability, %	F_4_	F_2_	2008	F	180	75.01	6.40	0.36	0.34	0.37
	F_7_	F_7_	2012	F	156	91.70	6.63	0.47	‐	‐
Panicle length, cm	F_2_	F_2_	2005	F	180	21.52	2.73	0.52	0.34	0.22
	F_4_	F_2_	2007, 2008	F	92	26.49	2.21	0.04	0.00	0.05
β‐glucan, %	F_7_	F_7_	2012	F	155	4.16	0.29	0.68	‐	‐
	F_7_	F_7_	2013	F	146	4.10	0.28	0.47	‐	‐
Grain area, mm^2^	F_7_	F_7_	2013	F	150	28.68	2.13	0.61	‐	‐

### 2.2 Genomic prediction models

Two genomic prediction methods were used: ridge regression‐BLUP (RR‐BLUP; Piepho, 2009) and differentially penalized regression (DiPR; Bentley et al., 2014; Ward, Rakszegi, Bedő, Shewry, & Mackay, 2015). The use of two methods allowed for validation of models, including testing the use of marker information in a single matrix against differential weighting of the dominant (DArT) and codominant (microsatellite and GBS) marker data combined using DiPR. The RR‐BLUP analysis used the package rrBLUP v4.6 (Endelman, 2011) in R v3.3.3 for Windows (R Core Team, 2016). Predictions were compared within a generation between lineages and between generations using an integrated data matrix. For the integrated data matrix, genotype data for dominant markers were attributed half scores to account for their uncertainty, akin to an imputed marker (i.e., AA or AB: 0.5; AB or BB: −0.5), whereas whole value scores were used for codominant marker data (i.e., AA: 1; AB: 0; BB: −1). Imputation of further missing marker data was performed using the random forest algorithm implemented with the R package missForest v1.4 (Stekhoven & Buhlmann, 2012) with 1,000 trees and using Chi‐squared tests for parameterization of the missForest model with artificially removed data. Five‐fold cross‐validation (CV) within generations was performed with 100 replications via Monte Carlo cross‐validation (MCCV; Xu & Liang, 2001). Cross‐validation between generations, between and within lineages, was performed with *k*‐fold CV (*k* = 2, 3, 4, and 5) to examine differential sampling depths from the available population (i.e., simulating a breeder having genotyped and phenotyped 0.50, 0.33, 0.25, or 0.20 of the early generation, respectively). Within generation predictions were made independently on Tall and Dwarf classes (as determined by F_2_ genotyping) to account for the known segregating *Dw6* gene (Molnar et al., 2012) when the training set size was greater than 30 individuals. In the full dataset, 27% of lines were classified as Tall.

All prediction accuracies are reported as pairwise Pearson correlations. In the within‐generation models, Fisher's Z‐transformation was used to convert Pearson correlations to a normal distribution (as Z is normally distributed whereas *r* is not) before averaging and back‐conversion. In the between‐generation models, where all available marker and phenotype data common to both generations were used to train and predict from the early generation (F_2_, F_3_ and F_4_) to RILs, accuracy is reported as the pairwise Pearson correlation within a family. In the between‐generation, between‐lineage models, genotypes were randomly sampled without replacement 100 times according to that *k*‐fold CV analysis (where *k* = 2–5). Accuracy is reported for both within and between family Fisher's Z‐transformed mean Pearson correlations with back‐conversion across the 100 per‐*k* iterations.

To implement DiPR, the common marker data from F_2_ to RIL genotypes were divided into dominant (DArT) and codominant (microsatellite and GBS) marker types. Markers were thinned at an *r^2^* value of .90 to prevent oversampling and an additive relationship matrix was derived for each marker type. These were linearly combined into a single matrix with separate weighting factors (w and 1−w), between w = 0 and w = 1 in 0.01 steps, to produce a single input to RR‐BLUP, as previously described (Bentley et al., 2014; Ward et al., 2015). Model fitting used the R package ‘RR‐BLUP’ (Endelman, 2011) and the optimal w‐value was determined as the maximum cross‐validation correlation. At w = 0, only the codominant markers contributed to the prediction and at w = 1, only the dominant markers contributed. The intervening weights use differential penalization consistent between matrices but with the two marker sets contributing to the additive relationship matrix proportional to their weighting (Bentley et al., 2014).

## 3 RESULTS

Across the early generation (F_2_) genotypes, there were 545 genotyped markers, of which 424 were dominant and 121 codominant. For the RIL (F_7_) population there were 1,046 codominant genotyped markers. Between the two datasets there were 401 common markers, of which 100 were codominant and 301 were dominant.

### 3.1 Within‐generation predictions

In order to determine the added value of early generation genotyping, within‐generation models were tested. Predictions were made using F_2_ genotype data to predict phenotypes at the F_2_, F_3_, and F_4_ level, while RIL genotype data (F_7_) was used to predict phenotypes at the RIL level (F_7_). A total of 15 phenotypes were predicted with 12 predicted at both the early and later RIL generation, and all data are presented in Table 1. At the F_2_ genotype level, all the phenotypes were compared across all lines as well as within *Dw6* genotypic Tall and Dwarf classes. The accuracy of prediction varied across traits and generations. The traits that were predicted to the highest levels of accuracy across generations were height (range .81–.89), ear emergence (.64–.79), and kernel content (.60–.66). For the majority of traits, the accuracy of prediction was higher when using F_7_ genotypic and phenotypic data compared to predicting in early generations (kernel content, maturity, mildew, panicle extrusion, grain yield, grain length, grain width, and hullability). Variation was observed for the accuracy of trait prediction when using different phenotyping generations or trial years for some traits including Internode 1 length (.34 from F_4_ compared to .79 from F_2_ and .83 from F_7_ phenotypes) and winter hardiness (.09 from F_3_, .47 in 2011 F_7_ trials to .70 from F_4_, and .77 from F_7_ in 2012 phenotypes). Predicting within *Dw6* classes gave generally low predictions for all traits when compared to predicting across the full dataset, with the exception of height, grain length, and width in the F_4_ and the overall low prediction traits (maturity, mildew, winter hardiness, and panicle length).

### 3.2 Between generation predictions

**FIGURE 2:**
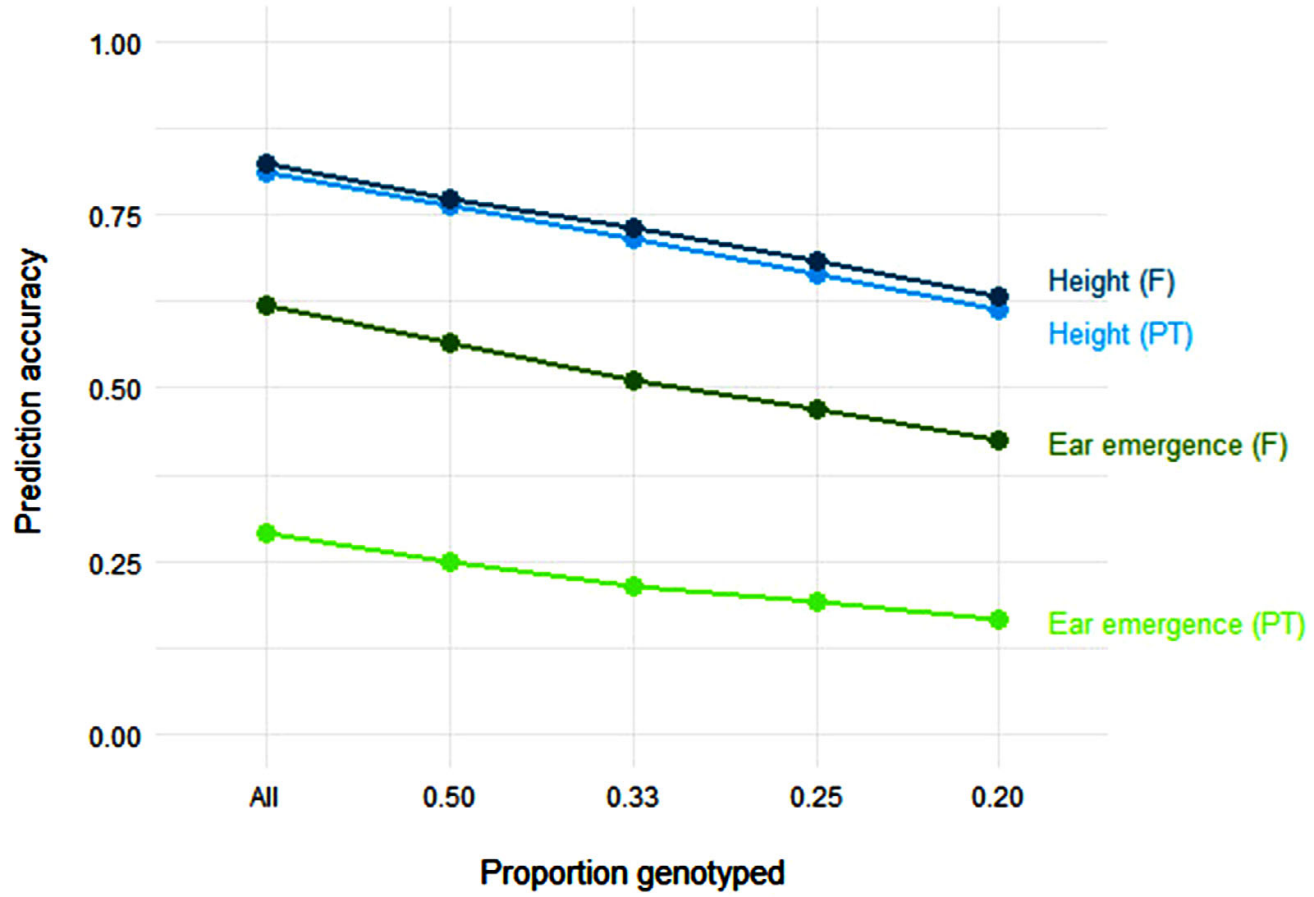
Comparison of changes in ridge regression best linear unbiased prediction accuracy from the F_2_ to F_7_ generation in ‘Buffalo’ × ‘Tardis’ recombinant inbred lines for height and ear emergence in the field (F) and polytunnel (PT) based on varying proportions of genotyped F_2_ individuals

To examine whether between‐lineage predictions were possible from early to late generations, predictions were made within and between lineages as well as across all available data. The phenotypic correlation between early generation and RIL phenotypes was used as a proxy for the accuracy of imposing selection on phenotype alone at the F_4_ generation for comparative purposes. Nine traits were selected for comparison to assess differences in predictive accuracy between the early and late generations and all data are presented in Table 2. In general, this showed that low‐level genotyping (combined with phenotyping) in the early generation was sufficient to allow relatively accurate predictions to be made for later generation (F_7_) phenotypes with the exception of kernel content (.66 for all markers, dropping to .39 with 50% of individuals). High prediction accuracies were maintained for Internode 1 length for predictions from the F_2_ to F_7_ (.58–.76) and F_4_ to F_7_ (.54–.77) across genotyping coverage as well as for panicle extrusion, grain yield, and height (Table 2). Where predictions overall were low (maturity, mildew, and winter hardiness), accuracies were maintained or slightly reduced with coverage. For ear emergence, the predictions from early generation to field grown F_7_ lines were high overall (.62) and showed a slow pattern of reduction with genotyping density, but predictions from early generations to F_7_ polytunnel phenotypes were low (.29 for F_2_, .12 for F_3_). This was not the case for height, with predictions stable across both field and polytunnel trials (Figure 2).

**TABLE 2:** Comparing within and between generation predictions in the ‘Buffalo’ × ‘Tardis’ population using training populations of early generation phenotyped (Gen^P^) individuals (Phen^n^) for common traits assessed in a test set composed of F_7_ recombinant inbred lines (RILs) in either the field (F) or polytunnel (PT). The true correlation between phenotypes (Corr^P^) is used as a proxy for early phenotypic selection. A comparison is made on the change in accuracy of prediction within and between generation when variable numbers of F_2_ individuals are genotyped (all including *Dw6* Tall and Dwarf class, 50, 33, 25, and 20%)

						Accuracy of prediction based on proportion of F_2_ individuals genotyped										
	Training set		Test set (F_7_)			Genotype all (F_2_)			Genotype 0.50		Genotype 0.33		Genotype 0.25		Genotype 0.20	
Trait	Gen^P^	Phen^n^	Trial	Phen^n^	Corr^P^	All	Tall	Dwarf	Within	Between	Within	Between	Within	Between	Within	Between
Internode 1 length	F_2_	161	PT	213	0.38	0.76	0.00	0.22	0.73	0.71	0.70	0.67	0.67	0.62	0.64	0.58
	F_4_	81	PT	213	0.77	0.77	0.30	0.21	0.75	0.69	0.74	0.65	0.72	0.59	0.72	0.54
Kernel content	F_4_	161	F	152	0.10	0.66	0.49	0.26	0.39	0.39	0.39	0.39	0.39	0.39	0.39	0.39
Maturity	F_4_	161	F	152	0.22	0.36	0.23	0.16	0.33	0.31	0.31	0.28	0.30	0.25	0.29	0.23
Mildew	F_3_	161	F	137	0.21	0.49	0.34	0.55	0.49	0.47	0.47	0.46	0.46	0.43	0.44	0.41
Panicle extrusion	F_4_	81	PT	213	0.86	0.82	0.21	0.00	0.81	0.73	0.81	0.69	0.80	0.64	0.80	0.60
Winter hardiness	F_3_	136	F	213	0.22	0.43	0.36	0.18	0.34	0.30	0.29	0.25	0.27	0.21	0.25	0.19
	F_4_	159	F	213	0.40	0.61	0.63	0.64	0.56	0.55	0.53	0.50	0.50	0.45	0.49	0.42
Grain yield	F_4_	161	F	213	0.38	0.63	0.03	0.25	0.57	0.53	0.52	0.46	0.48	0.40	0.46	0.36
Ear emergence	F_2_	161	PT	213	0.05	0.29	0.11	0.13	0.24	0.25	0.20	0.21	0.17	0.19	0.15	0.17
	F_3_	91	PT	213	0.04	0.12	0.00	0.00	0.00	0.12	0.00	0.08	0.00	0.06	0.00	0.05
	F_2_	161	F	213	0.19	0.62	0.15	0.00	0.56	0.57	0.51	0.51	0.47	0.47	0.44	0.42
	F_3_	91	F	213	0.54	0.62	0.03	0.00	0.53	0.57	0.52	0.51	0.51	0.47	0.50	0.44
Height	F_2_	161	PT	208	0.37	0.81	0.18	0.19	0.78	0.76	0.75	0.72	0.71	0.66	0.68	0.61
	F_3_	161	PT	208	0.50	0.86	0.11	0.05	0.84	0.82	0.81	0.78	0.78	0.73	0.75	0.68
	F_2_	161	F	208	0.37	0.82	0.04	0.46	0.79	0.77	0.75	0.73	0.73	0.68	0.68	0.63
	F_3_	161	F	208	0.50	0.87	0.20	0.05	0.84	0.83	0.81	0.78	0.78	0.74	0.76	0.69

### 3.3 Comparing methods for handling mixed marker data

Dominant markers provide less information than codominant markers but are cheaper to generate, meaning that a greater number are likely to be available (or required) to generate accurate predictions. The DiPR was implemented across nine traits (Table 3) to assess the predictive advantage of proportionally combining marker types in a single additive relationship matrix. When compared to predictions based on a single marker type using RR‐BLUP, the DiPR method performed as well or better than a RR‐BLUP model using a single matrix with results summarized in Table 3. Low RR‐BLUP predictions for maturity (.36), mildew (.49), and winter hardiness (F_3_ predictions .43) were all improved through the implementation of DiPR (.47, .52, and .48, respectively) although their optimal weighting factors varied. Differential weighting for these low‐prediction traits showed that only using codominant markers (DiPR w^opt^ = 0.00) improved maturity and winter hardiness predictions whereas using only dominant markers (w^opt^ = 1.00) optimized prediction of mildew.

**TABLE 3:** Comparison of methods to handle mixed dominant and codominant marker types for predictions in the ‘Buffalo’ × ‘Tardis’ population. The training population consists of early generation phenotyped (Gen^P^) individuals (Phen^n^) for common traits assessed in the test set composed of F_7_ recombinant inbred lines (RILs) in either the field (F) or polytunnel (PT). Prediction is compared between standard ridge regression best linear unbiased predictor (RR‐BLUP) and differentially penalized ridge regression (DiPR), with the associated optimal weight value (DiPR w^opt^) given

	Training set		Test set (F_7_)		Prediction accuracy		
Trait	Gen^P^	Phen^n^	Trial	Phen^n^	RR‐BLUP	DiPR	DiPR w^opt^
Internode 1 length	F_2_	161	PT	213	0.76	0.77	0.29
	F_4_	81	PT	213	0.77	0.76	0.93
Kernel content	F_4_	161	F	152	0.66	0.66	0.22
Maturity	F_4_	161	F	152	0.36	0.47	0.00
Mildew	F_3_	161	F	137	0.49	0.52	1.00
Panicle extrusion	F_4_	81	PT	213	0.82	0.84	0.03
Winter hardiness	F_3_	136	F	213	0.43	0.48	0.00
	F_4_	159	F	213	0.61	0.70	0.00
Grain yield	F_4_	161	F	213	0.63	0.69	0.23
Ear emergence	F_2_	161	PT	213	0.29	0.24	0.90
	F_3_	91	PT	213	0.12	0.09	0.42
	F_2_	161	F	213	0.62	0.60	0.68
	F_3_	91	F	213	0.62	0.63	0.27
Height	F_2_	161	PT	208	0.81	0.82	0.24
	F_2_	161	F	208	0.82	0.86	0.01
	F_3_	161	PT	208	0.86	0.85	0.26
	F_3_	161	F	208	0.87	0.87	0.05

## 4 DISCUSSION

As genotyping costs fall, there is an opportunity to use genomic prediction to reduce the number of individuals phenotyped in within‐cross breeding populations. We demonstrate that it is feasible to use the genotypic information from a full set of biparental lines to make within generation, between‐lineage genomic predictions. This can recover information on missing phenotypic data to improve selection resilience representing an added value to early generation genotyping beyond deselection of unfavorable alleles, as previously described for wheat (*Triticum aestivum*; He et al., 2016) and soybean (*Glycine max*; Ma et al., 2016). Early generation genotypic data can also be used to reduce the number of lines required in later generation phenotyping based on siblings progressed to generate stable, genotyped homozygous lines. Our results demonstrate that early generation genotyping need not cover the full population in order to attain accuracies in line with true trait correlation between early and late generation phenotypes (a proxy for selecting on early generation phenotypes alone), as has been previously shown in small populations (Wong & Bernardo, 2008). We therefore propose that strong within‐cross selection could be imposed early in a breeding cycle whilst retaining accuracy of selection. Prior simulations of within‐cross genomic prediction have been reported in maize, suggesting that gains plateau with selfing rounds, with the F_4_ capturing 90% of the F_8_ gains due to an increase in the maximal breeding value of the population (McClosky et al., 2013). If these gains can be identified within lineages in the early stages, then accurate selection could be imposed before phenotypic selection.

In this study we performed predictions with F_2_ and F_7_ (RIL) genotype data. In the first instance, F_2_ genotypes were employed to make models with early generation (F_2_, F_3_, and F_4_) phenotypes with 80% of available phenotype data as the training set and 20% as the test set. This simulates lost data in early generation phenotyping when an accurate representation of the cross’ phenotypic value is required for selection. A major limitation to the implementation of GS within small breeding programs is the high upfront genotyping cost (Varshney et al., 2012). Our results indicate that there is an advantage to early generation genotyping, and that this need not be at high coverage in order to provide value to between‐generation RIL performance prediction.

Prediction within the F_7_ RILs had generally high accuracy and demonstrates potential savings in later stage phenotyping costs. Where seed is generated for RIL phenotyping in a shuttle breeding framework (Borlaug, 1968; Forster et al., 2015), there is a requirement to transfer substantial quantities of seed between environments. Our data indicates that an alternative to the movement of large amounts of seed could be to use separate sets of families to be tested in multiple target environments and to use within‐generation prediction for the missing environment performance. However, this would need to be empirically tested as the effect of environmental variability on robustness of prediction are well documented (Burgueño, de los Campos, Weigel, & Crossa, 2012; Jarquín et al., 2014). This would be particularly attractive in Europe where out‐of‐season multiplication takes place in climatically matched environments in the southern hemisphere, representing a major cost. Using sets of small families could also enable the use of GS for adaptation to multiple target environments at an early stage in the breeding program. This is currently limited by seed availability and would have both cost and logistical advantages in using sibling predictions to avoid phytopathological quarantine requirements.

Our data indicate that between family predictions across generations could allow for earlier lineage selection. Early selection is currently limited due to high levels of heterozygosity and uncertain phenotypic value of lineages. However, if a portion of the F_2_ lineages are genotyped (as single plants), and their derived F_4_ field phenotypes (based on siblings from F_3_ rows) are used in conjunction with low‐coverage F_2_ genotyping, a genomic prediction model could be developed. Following subsequent production and genotyping of fixed RILs, a prediction can be used to select which of the cross’ lineages are likely to perform best and reduce the number of entries into fully replicated field trials, therefore accelerating the breeding cycle (Jannink, Lorenz, & Iwata, 2010). This offers the ability to use F_2:4_ families to predict F_7_s derived from different F_2_s and to rapidly generate F_7_ lines while producing a prediction equation over one or two seasons of yield testing. Selection among the F_7_ is then made on the predicted trait values. This theoretical program design is summarized in Supplemental Figure S1.

We also compared different proportions of F_2_ genotyping as an approximation for a breeder varying the level of financial investment in F_2_ genotyping, with all derived RILs then being genotyped. There was a reduction in predictive accuracy as the proportion of F_2_ genotyping declined although, even at low representation, some traits could still be predicted to the same levels as for phenotypic selection at the F_4_ generation. Similar results have previously been shown in biparental maize population simulations (Bernardo & Yu, 2007).

The employment of within‐cross predictions reported here must be tailored to the existing breeding program, particularly with respect to number of crosses per cycle and selection intensity in order to ensure financial viability. The evaluation of economic aspects of GS implementation are essential for wider application (Abed, Pérez‐Rodríguez, Crossa, & Belzile, 2018). However, given the ability to achieve rapid generation time (Watson et al., 2018), our accuracy results suggest that selections could be made much earlier, although this remains to be empirically tested within breeding programs. Given that between‐lineage accuracies are similar to within‐lineage accuracies, our data suggest that independent families can be used to predict across lineages. In addition to showing that between‐lineage prediction is possible, we also show that F_3_ and F_4_ segregated material (as used in shuttle breeding or remote testing) can be used to reduce the costs associated with multi‐environment testing. Bekele et al. (2018) recently demonstrated heading date prediction accuracies of up to .67 in independent training and test populations. The accumulation of data from many crosses also represents a first step to the full implementation of GS within a program (Gorjanc et al., 2017a; Sverrisdóttir et al., 2017; Edwards et al., 2019). However, we note that further work is required to compare the within‐cross predictions reported here to wider performance across a breeding program with analysis of all crosses jointly (Jannink et al., 2010). The longer‐term adoption and implementation of a multi‐subpopulation training population (de Roos, Hayes, & Goddard, 2009) offers an attractive gradual adoption model for GS in small programs if LD can be maintained with higher marker densities (Asoro et al., 2011).

Finally, we demonstrate that the use of mixed marker data can be optimized using DiPR. Although dominant marker use is declining, they still represent the cheapest genotyping method for low‐resource crops and much legacy data exists. Dominant markers are less informative than codominant markers and their use can be problematic for GS across generations because of varying levels of heterozygosity that cannot be accounted for. We considered an alternative to a linear combination of dominant and codominant markers that separately weighted marker types as components of a single additive relationship matrix. Implemented as DiPR (Bentley et al., 2014; Ward et al., 2015), this showed that for some traits an optimized weighted combination of the two marker types improved prediction accuracy compared to a combined matrix using all available data. When the weight factor (w) was zero, only codominant data was used in the prediction. As the weight tends toward one, more weight is applied to the dominant marker data. For example, for kernel content (training: F_4_ 2007, 2008, 2010; test: RIL 2012) an intermediate optimal solution (w^opt^ = 0.22) was found. This compares to mildew (training: F_3_ 2006; test: RIL 2012) which had a dominant marker optimum (w^opt^ = 1.00) and winter hardiness (training: F_3_ 2006 and F_4_ 2007; test: RIL 2012) which had a codominant marker optimum (w^opt^ = 0.00). Although dominant markers have been largely superseded by SNP‐based methods of genotyping, our results indicate that for some traits they provide useful information. The low frequency or uneven distribution of SNP markers across the oat genome (Bekele et al., 2018) may explain why the dominant markers used here made higher, or complete contributions to optimal predictions for disease (typically a dominant genetic effect, controlled by a limited number of loci; Okoń & Ociepa, 2018). Conversely, winter hardiness was optimally predicted from codominant markers and it is a documented complex, quantitative trait that has limited tractability in oat breeding programs (Chawade et al., 2012). Therefore, we propose that the genetic architecture of a trait combined with marker coverage are determinants of optimal DiPR weighting.

Our findings are potentially useful for other studies looking to combine data types in predictions. Asoro et al. (2013) previously proposed the use of selection criteria to weight low‐frequency favorable alleles in GS to avoid loss of diversity with increasing gains for β‐glucan in oat breeding. We also demonstrate the utility of within‐ and between‐generation predictions in a narrow‐base oat population. The predictions reported here would have benefits to a breeding program where genotyping costs are less than field trial costs. In this study we use a modest number of individuals and markers, but scaling to higher density markers, larger numbers of individuals, and broadening the population base are all opportunities for achieving future breeding gains.

## CONFLICT OF INTEREST

The authors declare no conflicts of interest.

## Supporting information

Supplemental Table S1. Phenotypic traits assessed in the ‘Buffalo’ × ‘Tardis’ population in this study including trait name and method used for assessment.
Supplemental Figure S1. Theoretical program design to increase selection pressure within a cross using genomic prediction based on development of a predictive model from F_2_ genotypes and F_4_ phenotypes applied to estimate between lineage performance in recombinant inbred lines (RILs). We propose that this scheme would permit stronger selection within cross without a loss of accuracy.

## Data Availability

The raw data used for the analysis reported in this study is available from the Triticeae Toolbox oat platform (http://triticeaetoolbox.org/oat/genotyping).
