# Supplementary material for: Implementing within‐cross genomic prediction to reduce oat breeding costs
Source: Plant Genome. 2020 Mar 17;13(1):e20004. doi: 10.1002/tpg2.20004 (PMC8638661; doi:10.1002/tpg2.20004)
Supplement: Supplementary file 1 — Supplemental Table S1. Phenotypic traits assessed in the ‘Buffalo’ × ‘Tardis’ population in this study including trait name and method used for assessment. Supplemental Figure S1. Theoretical program design to increase selection pressure within a cross using genomic prediction based on development of a predictive model from F2 genotypes and F4 phenotypes applied to estimate between lineage performance in recombinant inbred lines (RILs). We propose that this scheme would permit stronger selection within cross without a loss of accuracy. [file TPG2-13-e20004-s001.docx]

**SUPPLEMENTAL MATERIALS**

**Supplementary Table S1.** Phenotypic traits assessed in the ‘Buffalo x Tardis’ population in this study including trait name and method used for assessment.

| **Trait** | **Method of assessment** |
| --- | --- |
| Internode 1 length | Length (cm) from the panicle basal node to the next node below (node 1) |
| Kernel content | Determined by passing 25 g of whole grain through a Laboratory Oat Huller (Codema Model LH5095; Maple Grove, Minneapolis, USA) set at 100 bar for 60 seconds and then separating the output into groats and whole grain. Kernel Content (%) calculated as (Groat weight (g) / (Initial weight (g) – Whole grain weight (g)) x 100 |
| Maturity | Days from 1^st^ April to GS92 (Zadoks *et al.*, 1974) |
| Mildew | Scored in early spring on a 1-9 scale with 9 representing no damage (AHDB, 2012) |
| Panicle extrusion | Length (cm) from the flag leaf ligule to the panicle basal node |
| Winter hardiness | Scored in early spring on a 1-9 scale with 9 representing no damage (AHDB, 2012) |
| Grain yield | Grain harvested using a small plot combine and grain yields were adjusted to 15% moisture content |
| Ear emergence | Days from 1^st^ April to 50% of ears emerged in each plot (GS55; Zadoks et al., 1974) |
| Height | Scored immediately prior to harvest (5 measurements per plot in cm from ground level to the tip of the panicle) |
| Grain length | Determined using a MARVIN grain analyser (GTA Sensorik GmbH, Germany) |
| Grain width | Determined using a MARVIN grain analyser (GTA Sensorik GmbH, Germany) |
| Hullability | Determined from kernel content determination as 100 – (100 x Whole grain weight (g)/ Initial weight (g)) |
| Panicle length | Distance in cm from the basal panicle node to the tip of the panicle (5 plants per plot) |
| β-glucan | Determined on a subsample of ground groat using the McCleary method Megazyme™ kit K-BGLU (Megazyme International Ireland Ltd., Wicklow, Ireland). |
| Grain area | Determined using a MARVIN grain analyser (GTA Sensorik GmbH, Germany) |

**References**

AHDB (2012) Recommended list protocol. Available from: <https://ahdb.org.uk/knowledge-library/recommended-lists-protocols>

Zadoks JC, Chang TT, Konzak CF (1974) Decimal code for growth stages of cereals. Weed Research 14, 415-421.

**Supplementary Figure S1**. Theoretical programme design to increase selection pressure within a cross using genomic prediction based on development of a predictive model from F2 genotypes and F4 phenotypes applied to estimate between lineage performance in recombinant inbred lines (RILs). We propose that this scheme would permit stronger selection within cross without a loss of accuracy.

Parent A x Parent B

F_2_ plants

(single plant genotyping)

RIL replicated trial

F_4_ replicated trial (phenotyping F_4_)

SSD

F_3_ rows

Siblings

RIL

(single plant genotyping)

SSD

*Develop predictive model PM from F_2_ genotypes and F_4_ phenotypes*

*Apply PM to estimate between lineage performance*
